# Quercetin and Green Tea Extract Supplementation Downregulates Genes Related to Tissue Inflammatory Responses to a 12-Week High Fat-Diet in Mice

**DOI:** 10.3390/nu9070773

**Published:** 2017-07-19

**Authors:** Lynn Cialdella-Kam, Sujoy Ghosh, Mary Pat Meaney, Amy M. Knab, R. Andrew Shanely, David C. Nieman

**Affiliations:** 1 Department of Nutrition, School of Medicine—WG 48, Case Western Reserve University, 10900 Euclid Avenue, Cleveland, OH 44106, USA; lynn.kam@case.edu; 2 Program in Cardiovascular & Metabolic Diseases and Center for Computational Biology, Duke NUS Medical School, 8 College Road, Singapore 169857, Singapore; sujoy.ghosh@duke-nus.edu.sg; 3 Department of Exercise Physiology, School of Health Sciences, Winston-Salem State University, 601 S. Martin Luther King Jr. Drive, Winston-Salem, NC 27110, USA; meaneyMP@wssu.edu; 4 Levine Center for Health and Wellness, Queens University of Charlotte, 1900 Selwyn Avenue, Charlotte, NC 28274, USA; knaba@queens.edu; 5 Department of Health & Exercise Science, Appalachian State University, ASU Box 32071, 111 Rivers Street, 050 Convocation Center, Boone, NC 28608, USA; shanelyra@appstate.edu; 6 Human Performance Laboratory, North Carolina Research Campus, Appalachian State University, 600 Laureate Way, Kannapolis, NC 28081, USA

**Keywords:** cytokines, fat metabolism, flavonoids, inflammation, insulin resistance, immune function, obesity, metabolic syndrome, phytochemicals

## Abstract

Quercetin (Q) and green tea extract (E) are reported to counter insulin resistance and inflammation and favorably alter fat metabolism. We investigated whether a mixture of E + Q (EQ) could synergistically influence metabolic and inflammation endpoints in a high-fat diet (HFD) fed to mice. Male C57BL/6 mice (*n* = 40) were put on HFD (fat = 60%kcal) for 12 weeks and randomly assigned to Q (25 mg/kg of body weight (BW)/day), E (3 mg of epigallocatechin gallate/kg BW/day), EQ, or control groups for four weeks. At 16 weeks, insulin sensitivity was measured via the glucose tolerance test (GTT), followed by area-under-the-curve (AUC) estimations. Plasma cytokines and quercetin were also measured, along with whole genome transcriptome analysis and real-time polymerase chain reaction (qPCR) on adipose, liver, and skeletal muscle tissues. Univariate analyses were conducted via analysis of variance (ANOVA), and whole-genome expression profiles were examined via gene set enrichment. At 16 weeks, plasma quercetin levels were higher in Q and EQ groups vs. the control and E groups (*p* < 0.05). Plasma cytokines were similar among groups (*p* > 0.05). AUC estimations for GTT was 14% lower for Q vs. E (*p* = 0.0311), but non-significant from control (*p* = 0.0809). Genes for cholesterol metabolism and immune and inflammatory response were downregulated in Q and EQ groups vs. control in adipose tissue and soleus muscle tissue. These data support an anti-inflammatory role for Q and EQ, a result best captured when measured with tissue gene downregulation in comparison to changes in plasma cytokine levels.

## 1. Introduction

High-fat Western diets are associated with insulin resistance, inflammation, and de novo lipogenesis [1,2], which are factors that contribute to the development of metabolic syndrome. Flavonoid ingestion has the potential to partially offset these effects. In particular, quercetin and epigallocatechin gallate (EGCG) from green tea have been reported to attenuate insulin resistance, counter inflammation, and favorably alter fat metabolism [2,3,4,5]. However, the effect of a mixture of quercetin and EGCG has been examined in only a few studies.

Quercetin is a flavonoid that is found in many plant and foods such as onions, green tea, apples, peppers, and berries [6]. Both in vitro and rodent models provide evidence that quercetin supplementation reduces various measures related to metabolic syndrome [2,3,7]. Specifically, quercetin has been reported to blunt pro-inflammatory signaling via regulation of NF-κβ-associated mechanisms in adipocytes, macrophages, and other cell lines [8,9,10,11,12,13], decrease insulin intolerance in primary human adipocytes and 3T3-L1 cells [8,14], and inhibit adipogenesis in 3T3-L1 cells [14,15,16] and lipid body formation in macrophages [17]. In rodents, quercetin has been reported to lower levels of circulating inflammatory-related plasma cytokines [18], inhibit pro-inflammatory signals [11,19,20,21], and improve insulin sensitivity [20,21,22,23,24,25,26,27] and dyslipidemia [20,21,24,26,27,28]. Very few human studies have examined the relationship between quercetin supplementation and metabolic syndrome risk factors in overweight adults. In a double-blinded, placebo-controlled study, Egert et al. [29] reported that six weeks of supplementation of quercetin at 150 mg/day reduced systolic blood pressure and plasma oxidized low-density lipoprotein (LDL) concentrations in overweight adults (*n* = 93; mean age = 45.1 years), but had no effect on inflammation. However, the effect of quercetin supplementation on lipid markers appears to vary based on apolipoprotein (APOE) genotype. Similarly, six weeks of onion-extract supplementation (quercetin of 162 mg/day) was associated with a reduction in 24-h ambulatory blood pressure in overweight/obese adults (*n* = 68, mean age = 47.4 years) with central obesity and pre-hypertension [30]. However, quercetin supplementation had no impact on endothelial function, inflammation, oxidative stress, and lipid and glucose metabolism in these individuals [30]. In large community studies including both normal weight and overweight female adults, quercetin supplementation at 500 mg/day or 1000 mg/day for 12 weeks was reported to have no influence on innate immune function or inflammation [31], body composition [32], or disease risk factors [33]. Quercetin supplementation was, however, associated with a reduction in the severity and number of sick days associated with upper respiratory tract infections (URTI) in adults [34]. To our knowledge, only two studies have examined the influence of quercetin supplementation on insulin sensitivity. In one study, a 17.5% improvement in the homeostatic model assessment of insulin resistance (HOMA-IR) was reported in women with polycystic ovary syndrome (PCOS; *n* = 82, age = ~30 years) after 12 weeks of quercetin supplementation (1000 mg/day) [35]. In contrast, four weeks of quercetin supplementation (500 mg/day) had no impact on fasting blood glucose levels in healthy males (*n* = 22, age = 29.9 years) [36].

EGCG, a catechin, is the most abundant flavonoid found in green tea [6] and has been reported to have anti-obesity, anti-diabetic, and anti-inflammatory properties [2,3,37]. Notably, in vitro studies indicate that EGCG suppressed insulin resistance [38,39] and promoted glucose uptake via enhanced GLUT4 translocation [39,40] in skeletal muscle cells, attenuated β-cell release of insulin from mouse and human islet cells [39], and improved insulin sensitivity in human hepatocytes (HepG2 cells) [41]. Furthermore, EGCG was associated with decreased glucose uptake [42], lipid accumulation [43,44,45], adipogenesis [46], and adipocyte differentiation [44] in 3T3-L1 adipocytes, and reduced inflammation by reactive oxygen species generation in macrophages [47]. In rodents, EGCG and green tea extract have been shown in most studies to reduce total body and adipose tissue weights [37,48,49], decrease blood/plasma glucose and insulin levels [37,48,50], improve insulin sensitivity [37,48], blood pressure, and lipid profile [37,48,51], and reduce unfavorable obesity-associated changes in gut microbiota [52]. Epidemiological research and meta-analyses in general support the anti-obesity and health effects of EGCG [53]. In randomized controlled studies in humans, three studies found a small but significant decrease in body weight, waist circumference, and body fat with green tea supplementation [54,55,56], while two studies found no change [57,58]. Several meta-analyses of randomized controlled trials with green tea indicate a possible reduction in blood pressure [59,60,61], total and low-density lipoprotein cholesterol [60,62,63], and fasting blood glucose and insulin insensitivity [64].

Given the independent effects of quercetin and EGCG on metabolic syndrome, we aimed to elucidate whether the combined effort of quercetin and green tea extract supplementation would improve blood glucose tolerance, decrease inflammation, and favorably alter metabolism in mice fed a high-fat diet. Previous studies by our research group suggest that ingestion of both quercetin and EGCG-enriched green tea extract have a greater anti-inflammatory effect than quercetin alone [65,66,67,68]. We utilized whole genome transcriptome and real-time polymerase chain reaction (qPCR) analysis of adipose, liver, and skeletal muscle tissues in mice fed high-fat diets to improve our ability to measure potential metabolic and anti-inflammatory effects related to flavonoid ingestion.

## 2. Materials and Methods

### 2.1. Animals and Experimental Design

**Figure 1:**
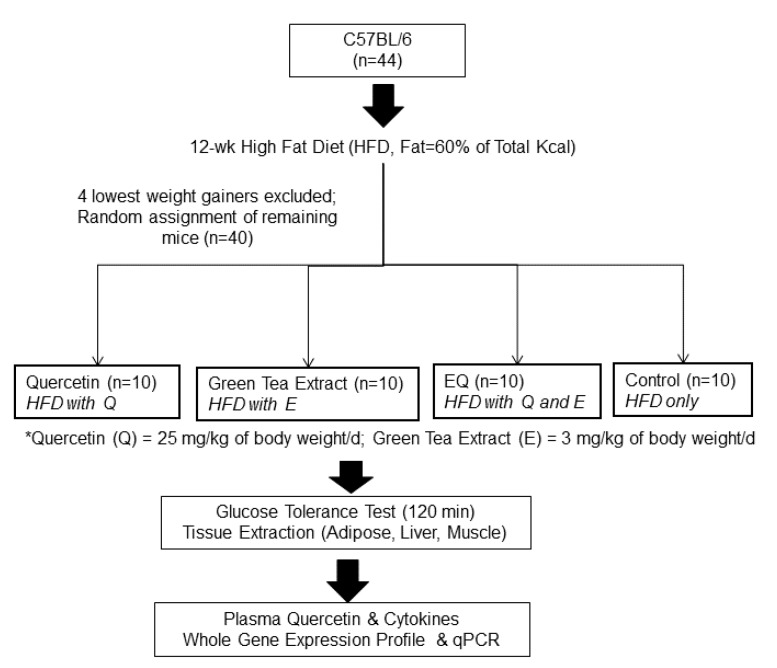
Study Design: C57BL/6 mice (*n* = 40) were placed on a high-fat diet (fat = 60% of total kcal) for 12 weeks and then randomly assigned to a diet supplemented with quercetin only (Q), green tea extract only (E), quercetin + green tea extract (EQ), or control (i.e., high fat diet only) for four weeks. The quercetin dosage was 25 mg of quercetin/kg of body weight (BW) per day, and green tea extract dosage was 3 mg of epigallocatechin gallate/kg BW per day.

Forty C57BL/6 mice (male, 5 weeks old, *n* = 44), purchased from a commercial vendor (Jackson Laboratory, Bar Harbor, ME, USA), were provided ad libitum access to a high-fat diet (HFD, fat = 60% kcal; BioServ, Frenchtown, NJ, USA) and water and maintained in 12 h light/dark cycle for the first 12 weeks at the animal facility of the North Carolina Research Campus. The experimental design is depicted in Figure 1. After 12 weeks on HFD, the four mice with the least weight gain were excluded from the second phase of the study, and the remaining mice (*n* = 40) were randomly assigned to one of four treatment groups (*n* = 10 per group): quercetin only (Q, 25 mg/kg of body weight (BW)/day of quercetin), green tea extract only (E; 3 mg/kg BW/day of EGCG), quercetin and green tea extract (EQ; 25 mg/kg BW of quercetin plus 3 mg/kg of EGCG), or control. All mice were maintained on HFD and with the exception of the control group were also supplemented with Q, E, or both for four weeks. Body weight was monitored weekly. At 16 weeks, mice underwent a glucose tolerance test and then were sacrificed. Tissue and plasma samples were collected for further analysis (Figure 1). All protocols utilized were approved by The Institutional Animal Care and Use Committee (IACUC) of the North Carolina Research Campus.

### 2.2. Glucose Tolerance Test and Blood and Tissue Collection

Following the four-week treatment period, mice fasted for 14 h and then were anesthetized and placed on a warming blanket. Next, mice were injected intraperitoneally with 2 g of glucose/kg BW. Blood (~3 μL) was collected from the tail vein, and blood glucose levels were measured at 0, 15, 30, 60 and 120 min using OneTouch Ultra^®^ blood glucometer (LifeScan, Johnson & Johnson, Chesterbrook, PA, USA).

Upon completion of the glucose tolerance test, mice were sacrificed, and whole blood was collected by cardiac puncture and centrifuged at 1000× *g* for 10 min at 4 °C. Plasma samples were aliquoted, snap frozen in liquid nitrogen, and stored at −80 °C for later analysis. The following tissue was harvested from the mice: left lobes of kidney and liver, pancreas, visceral adipose, subcutaneous adipose, and skeletal muscle tissue (soleus, gastrocnemius, plantaris, EDL, and quadriceps). All tissue was weighed. Tissue was either stored in RNAlater^TM^ (ThermoFischer Scientific, Waltham, MA, USA) per manufacturer’s instructions for genomics or frozen in liquid nitrogen and stored at −80 °C for later analysis.

### 2.3. Biochemical Assays

Plasma samples were pooled to assess quercetin, which was measured following solid-phase extraction via reversed-phase high-performance liquid chromatography with UV detection as previously described [65,66,67,68]. Plasma cytokines (IFN-γ, IL-1β, IL-6, IL-10, KC/GRO/CINC, and TNF-α) were measured using Mouse ProInflammatory 7-Plex Base Kit (Meso Scale Discovery, Rockville, MD, USA) per manufacturer’s instructions.

### 2.4. Genomic Analysis

Whole genome expression profiling was conducted with total RNA isolated from adipose, liver and skeletal muscle from mice in the Q, EQ and control groups. RNA was isolated and quantified, and quality control (QC) was performed on all samples. Expression profiling was performed on Mouse ST 1.1 PEG array (Affymetrix, ThermoFischer Scientific, Waltham, MA, USA) as per the manufacturer’s instructions. Signal extraction and background was subtracted for normalization utilizing Robust Multichip Average [69]. Samples that were considered outliers were excluded based on the QC report and scatter plots. Both the mean signal per treatment group and fold-change (log ratio) were calculated. CyberT was used to identify differentially expressed genes [70]. Pathways affected by each treatment relative to the control was determined using overrepresentation analysis via Ingenuity Pathway Analysis (IPA) software (Qiagen, Redwood City, CA, USA).

To quantify the expression of individual genes (*n* = 27), qPCR was performed in tissue samples from fat, liver, and soleus for the four experimental groups using Applied Biosystems™ TaqMan^®^ Gene Expression Assays (ThermoFischer Scientific, Waltham, MA, USA) as per the manufacturer’s instructions. Genes examined include those involved in cholesterol regulation (Abca1, Apoa1, Cyp3a41a, Srebf1, and Srebf2), fatty acid metabolism (Lpl, Ppara, Pparag. and Scd1), inflammatory and immune response (Cc12, Cd68, Ikbkb, Il1r1, Nfkb1, and Nr1h3), adipokines (Adipoq and Lep), oxidative stress (Ppargc1a), stress response (Hspa1a, Hspa2, Mapk8, and Sirt1), transcription (Atf2 and Nfact3), and xenobiotics (Cyp2e1).

### 2.5. Statistical Analysis

Data was summarized using means and standard error. To detect significant differences between groups, one-way ANOVA (time × treatment) was used for blood analysis and gene expression. Whole-genome expression profiles were examined via gene-set enrichment analysis (GSEA) [71]. A *p*-value was set at <0.05 for significance. Analysis was conducted using SAS 9.3 (SAS Institute, Cary, NC, USA).

## 3. Results

### 3.1. Body Mass and Biochemical Analysis

**Figure 2:**
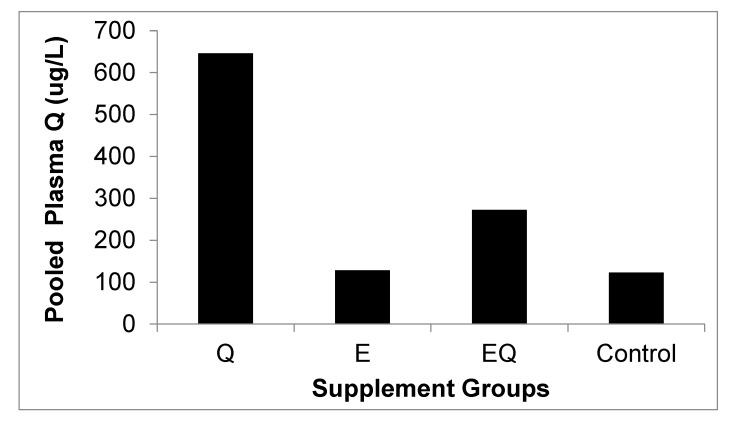
Pooled plasma quercetin at 16 weeks by experimental groups. C57BL/6 mice (*n* = 40) were placed on a high-fat diet (fat = 60% of total kcal) for 12 weeks and then randomly assigned to a diet supplemented with quercetin only (Q), green tea extract only (E), quercetin + green tea extract (EQ), or control (i.e., high-fat diet only) for four weeks. The dosage for quercetin was 25 mg of quercetin/kg of body weight (BW) per day and green tea extract dosage was 3 mg of epigallocatechin gallate/kg BW per day. Plasma samples were pooled for each group and analyzed for quercetin. At 16 weeks, plasma quercetin levels were 525% higher in Q, and 225% higher in EQ compared to control.

**Figure 3:**
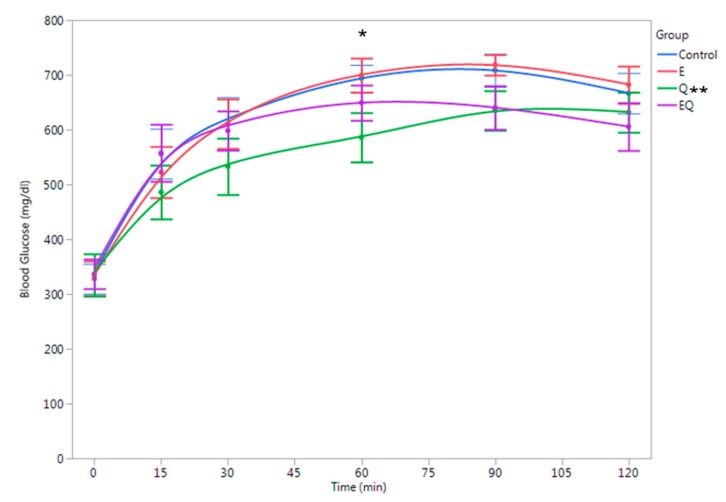
Glucose tolerance curve at 16 weeks by supplement groups. C57BL/6 mice (*n* = 40) were placed on a high-fat diet (fat = 60% of total kcal) for 12 weeks and then randomly assigned to a diet supplemented with quercetin only (Q), green tea extract only (E), quercetin + green tea extract (EQ), or control (i.e., high-fat diet only) for four weeks. The dosage for quercetin was 25 mg of quercetin/kg of body weight (BW) per day and green tea extract dosage was 3 mg of epigallocatechin gallate/kg BW per day. * Q lower than control at 60-min (*p* < 0.05). ** Area-under-the-curve (AUC) estimations lower for Q vs. EQ (*p* < 0.05).

**Figure 4:**
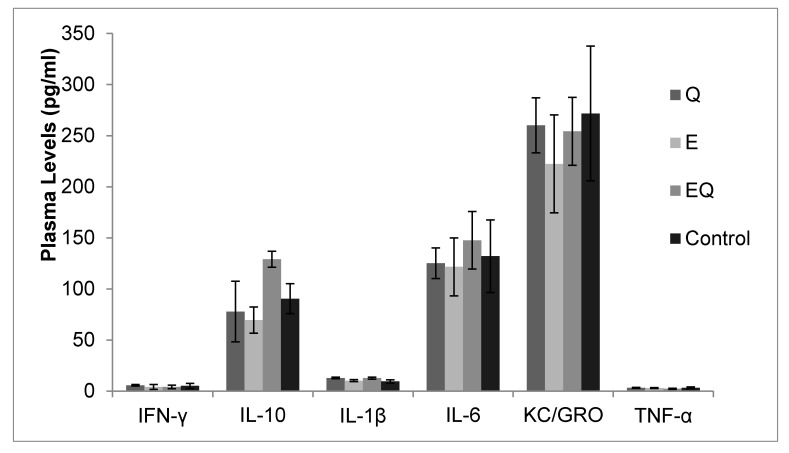
Plasma cytokine levels at 16 weeks by supplement groups. C57BL/6 mice (*n* = 40) were placed on a high-fat diet (fat = 60% of total kcal) for 12 weeks and then randomly assigned to a diet supplemented with quercetin only (Q), green tea extract only (E), quercetin + green tea extract (EQ), or control (i.e., high fat diet only) for four weeks. The dosage for quercetin was 25 mg of quercetin/kg of body weight (BW) per day and green tea extract dosage was 3 mg of epigallocatechin gallate/kg BW per day. Plasma cytokine levels did not differ between supplement groups and control (*p* > 0.05).

At the beginning of the study, the body mass for all mice was 20.0 ± 0.0 g with no differences among groups (*p* > 0.05). Body mass was also similar among groups at 12 weeks (*Q* = 47.3 ± 0.7 g, *E* = 47.1 ± 0.8 g, EQ = 47.1 ± 0.8 g, and control = 47.1 ± 1.0 g; *p* > 0.05) and at 16 weeks (i.e., after four weeks of supplementation (*Q* = 51.1 ± 0.6 g, *E* = 50.6 ± 0.8 g, EQ = 50.5 ± 0.5 g, and control = 50.2 ± 0.7 g; *p* > 0.05). At 16 weeks, pooled plasma quercetin levels were ~fivefold higher in Q and twofold higher in the EQ group compared to the control group (Figure 2). Glucose tolerance test (GTT) results are presented in Figure 2. Area-under-the-curve (AUC) estimations for plasma glucose were 14% lower for Q vs. EQ (*p* = 0.031) and trended 11% lower than control, but did not reach significance (*p* = 0.081). Plasma glucose was lower for Q vs. control at 60 min (*p* = 0.032; Figure 3). No other differences among groups were detected (*p* > 0.05; Figure 3). Plasma cytokines levels were also similar among groups (*p* > 0.05, Figure 4).

### 3.2. Genomic Analysis

**Figure 5:**
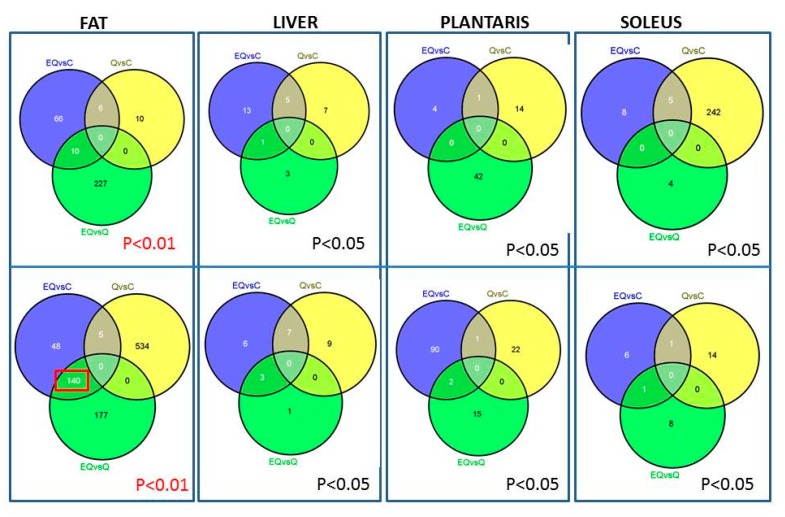
Overlap among differential expressed genes by tissue. Top panel shows the number of downregulated genes and the bottom panel shows upregulated genes. C57BL/6 mice (*n* = 40) were placed on a high-fat diet (fat = 60% of total kcal) for 12 weeks and then randomly assigned to a diet supplemented with quercetin only (Q), green tea extract only (E), quercetin + green tea extract (EQ), or control (i.e., high fat diet only) for four weeks. The dosage for quercetin was 25 mg of quercetin/kg of body weight (BW) per day, and green tea extract dosage was 3 mg of epigallocatechin gallate/kg BW per day. EQ treatment result in the upregulation of 140 genes compared to the control and Q groups.

Both microarray and IPA analysis revealed downregulation of genes associated with cholesterol metabolism and immune/inflammation in adipose tissue and soleus muscle tissue, fatty acid metabolism in soleus muscle tissue, and CYP450 metabolism in the liver. EQ resulted in downregulation of over 100 genes in adipose tissue compared to both control and quercetin alone (*p* < 0.01; Figure 5). The specific pathways downregulated by EQ and Q are depicted in Table 1. In skeletal muscle, protein ubiquination, the pathway responsible for marking proteins for degradation, was upregulated by Q treatment relative to the control.

In Table 2, gene expression changes are presented related to the plasma cytokines assessed. Of these, KC/GRO (i.e., Cxcl1) gene was expressed in adipose tissue and liver with an upregulation of KC/GRO detected in the liver of the EQ group compared to control (Table 2). The II-1β gene was also expressed in the liver, but no difference was found among treatments (Table 2). Q was associated with the downregulation of the II-1β receptor gene in adipose (Table 2) vs. control, and a downregulation trend was observed for other cytokine receptors genes in adipose tissue and soleus muscle tissue (*p >* 0.05, Table 1). For the EQ treatment, the IL-10 receptor gene was downregulated while the TNF-α receptor gene was upregulated in comparison to the control (Table 2). No differences were detected between Q and EQ groups for the genes presented in Table 2.

Of the 27 individual genes evaluated in adipose, soleus, and liver via qPCR, Q was associated with downregulation of three genes in adipose tissue, and no gene changes in the soleus or liver tissue compared to the control group (Table 3). In the soleus tissue, EQ and Q were associated with the downregulation of genes (4 and 2 genes, respectively) in the soleus vs. control with no other changes observed in adipose or liver tissue (Table 3).

## 4. Discussion

In mice on a 12-week HFD, four weeks of EQ supplementation were associated with the downregulation of over 100 genes in adipose tissue, including those involved in phagocytosis and leukocyte extravasation or trafficking pathways. Recruitment of leukocytes, specifically neutrophils, to adipose has been implicated in chronic inflammation in adipose tissue [72,73] and has been linked to insulin resistance in mice on HFD [73]. Traditional biomarkers for inflammation and glucose tolerance, however, were not different between EQ and control groups, but a mild improvement in blood glucose tolerance was detected with the Q treatment. In adipose and muscle tissue, EQ was associated with a downregulation of cholesterol metabolism compared to control. Cholesterol accumulation in adipose and muscle tissue have been associated with obesity and sarcopenia [74,75]. Genes associated with drug metabolism were also downregulated in EQ vs. control in the liver. The implications, however, are unclear, as changes in drug metabolism vary by metabolic and excretion pathways in obese individuals [76]. Thus, four weeks of EQ supplementation in mice on a 12-week HFD resulted in changes in tissue gene expression suggestive of reduced inflammation and cholesterol metabolism, while blood markers of glucose tolerance and inflammation were largely unaltered.

In the EQ group, the changes in tissue gene expression are indicative of reduced inflammation and leukocyte trafficking, which has been examined as a treatment target for inflammatory diseases [77]. Cytokine levels in the present study were not different among the experimental groups. Our findings in mice (~age in human = 50 years) [78] parallel previous studies in middle-aged humans. In overweight and obese women (*n* = 48, age = 56 years), 10 weeks of supplementation with mixed flavonoid-nutrient-fish oil supplement (Q-mix; 1000 mg quercetin, 400 mg isoquercetin, 120 mg EGCG, 220 mg EPA, and 180 mg DHA, 1000 mg vitamin C, 40 mg niacinamide, and 800 µg folic acid) did not alter biomarkers of inflammation, oxidative stress, and blood lipid levels, but was associated with gene alterations suggestive of enhanced antiviral defense and decreased leukocyte trafficking [79]. Similarly, in a randomized, double-blinded, crossover study in overweight men (*n* = 26, age = 46 years), Bakker et al. [78] reported no change in traditional biomarkers, but did report a shift in nutrigenomic profiles, which was associated with a reduction in inflammation after a five-week, anti-inflammatory dietary mix supplementation (AIDM, 6.3 mg resveratrol, 3.75 mg lycopene, ~38 mg EGCG, 300 mg EPA, 260 mg DHA,125 mg vitamin C, and 90.7 mg α-tocopherol) vs. placebo [80]. The relative dose of both quercetin and ECGC was higher in the present study compared to the human studies (*Q* = 25 mg/kg BW vs. AIDM = 0 mg/kg BW and Qmix = ~15 mg/kg BW; and *E* = 3 mg/kg BW vs. AIDM = ~0.4 mg/kg BW and Qmix = ~1.3 mg/kg BW). In addition, the respective duration of the supplementation was longer in the present study (i.e., four weeks of supplementation in mice is equivalent to ~10 years in human). Finally, the supplements in the human studies also contained fish oil, which has been associated with improvement in inflammatory biomarkers [81,82]. Taken together, these studies provide evidence that a mixture of flavonoids may be a promising treatment for reducing inflammation in overweight/obese individuals. Further research is needed to elucidate the optimal combination of flavonoids and/or whether the inclusion of fish oil in the supplementation provides additive benefits. Part of the challenge is the inclusion of novel outcome measures that capture metabolic and anti-inflammatory effects that are missed by basic plasma inflammation biomarkers [79]. The tissue-specific transcriptomic change observed in the present study may possibly reflect an earlier stage of tissue response to supplementation. Systemic changes may follow, and further research is thus warranted that examines a prolonged period of supplementation and/or supplementation at higher doses.

In the present study, plasma quercetin levels were lower in the EQ vs. Q group, despite the same dosage of quercetin being provided to both groups. Our findings are consistent with a mouse study conducted by Wang et al. [83], in which the authors reported that total quercetin levels in tissue were lower with the co-administration of EGCG and quercetin in mice. In the intestine, quercetin in humans is absorbed via passive diffusion as a primary route, and organic anion transporting polypeptide (OATPs) as a secondary route [84,85]. EGCG has been speculated to interfere with quercetin absorption via OATPs by acting as a non-competitive inhibitor or decreasing the activity of the transporter [83,86]. Given the high dose of Q administered in the present study, decreased absorption via OATPs could explain the lower plasma Q levels observed in the EQ group compared to Q and thus supporting the hypothesis of EGCG’s interaction with this transporter.

A mild improvement in blood glucose tolerance was associated with quercetin alone in this study. As previously discussed, very few human studies [35,36] have examined the impact of quercetin on blood glucose control and insulin resistance. Mehta et al. [82] reported that male Swiss albino mice (age not reported) had less stress-induced hyperglycemia and insulin-resistance following three weeks of quercetin supplementation (30 mg/kg) vs. control. Henagan et al. [87] reported that eight weeks of a low dose of quercetin (~1.6 g/kg BW) compared to a placebo resulted in improved insulin tolerance in male C57BL/6J mice (~14 weeks of age at sacrifice) on HFD, while the high dose (20 g/kg BW) did not alter insulin tolerance. The mice in the present study were older and had a higher dosage of quercetin compared to Henagan et al. [87]. In the EQ group, blood glucose levels were similar to both the control and E groups, but were higher than the Q group. As discussed previously, plasma quercetin levels were lower in the EQ vs. Q, which was possibly related to the interference of EGCG on quercetin’s absorption. Thus, the observed differences support quercetin’s role in improving glucose tolerance. A potential limitation in the current study is that the glucose tolerance test was conducted while the mice were under general anesthesia, which may have caused stress-induced hyperglycemia in all groups [88]. In addition, blood glucose was measured utilizing a glucometer, which has been reported to overestimate blood glucose levels in hyperglycemic states [89]. Thus, the measured blood glucose may have been higher than actual levels. Furthermore, it is difficult to separate hyperglycemia caused by the stress of anesthesia vs. HFD, and this may have confounded the potential impact of flavonoid supplementation on HFD-induced hyperglycemia.

Finally, the metabolism of flavonoids in human and mice differ, and more research is needed to determine the applicability of our results to human populations. The agreement between our prior human trial and the current mouse-based study indicating a downregulation in expression of genes related to leukocyte trafficking following mixed flavonoid supplementation is one indicator of similar responses between species [79]. In humans, polyphenols are transformed into metabolites with diminished biological impact [90,91,92]. Unabsorbed polyphenols can undergo bacterial bioconversion by gut microbiota into more bioactive forms [90,91,92]. Mice have different species of gut bacteria compared to humans, which limits the applicability of this model [92]. Humanized mice models have been suggested that utilize human fecal microbiota transplants (FMT) in mice to create a similar gut microbiome [92]. In addition, mice expressing the human drug metabolizing enzymes, cytochromes P450, may also prove to be a useful animal model in examining flavonoids [93]. Despite these differences, the plasma and urine content of quercetin metabolites are similar between humans and rats in type and number [94], and the bioavailability of EGCG have been reported as similar between human and mice [95]. Taken together, future studies on polyphenol mixtures could compare humanized and standard mouse models (e.g., those with FMT) to determine both similarities and differences on metabolic and inflammation outcome measures.

## 5. Conclusions

Supplementation with EQ for four weeks in mice fed a high fat diet for 12 weeks was associated with tissue gene expression changes suggestive of reduced inflammation and diminished leukocyte cell trafficking, a result we have previously demonstrated in human participants [79]. Traditional inflammatory biomarkers and glucose tolerance were not altered by EQ, but a mild improvement in glucose tolerance was observed with Q only. Future research should consider comparing flavonoid biotransformation in humanized mouse models to standard mouse models. Furthermore, lower doses and different flavonoid mixtures should be examined in both sedentary and physically active rodent models.

## Figures and Tables

**Table 1:** Top canonical pathways altered by four-week supplementation vs. control as identified by Ingenuity Pathway Analysis (IPA) ^1^.

Downregulated Pathways	Fat	Liver	Muscle	Comments
Steroid Biosynthesis		Q; EQ		Target of Statins
Phagocytosis/ leukocyte extravasation	EQ			Innate Immune Response
EIF2 signaling			Q; EQ	Stress Response
Mitochondrial dysfunction			Q	Associated with disease
eIF4/p70S6K signaling			Q; EQ	Insulin Signaling
Oxidative phosphorylation			Q; EQ	Energy Production
PPARα/RXRα activation			Q	Gene Expression

^1^ C57BL/6 mice (*n* = 40) were placed on a high-fat diet (fat = 60% of total kcal) for 12 weeks and then randomly assigned to a diet supplemented with quercetin only (Q), green tea extract only (E), quercetin + green tea extract (EQ), or control (i.e., high fat diet only) for four weeks. The dosage for quercetin was 25 mg of quercetin/kg of body weight (BW) per day and green tea extract dosage was 3 mg of epigallocatechin gallate/kg BW per day. IPA analysis was only conducted on tissue collected from the EQ, Q and control groups.

**Table 2:** Fold change in genes associated with cytokines assessed in plasma vs. control, based on microarray analysis ^1^.

Description	*Q*	*p*	EQ	*p*	Description
	Change		Change		
Adipose					
Ifngr1	–0.28	0.340	–0.18	0.659	interferon gamma receptor 1
Ifngr2	–0.14	0.820	–0.54	0.090	interferon gamma receptor 2
Il10ra	–0.36	0.550	–0.63	0.194	interleukin 10 receptor, alpha
Il10rb	–0.29	0.550	–0.73	0.047	interleukin 10 receptor, beta
Il1r1	–060	0.037	–0.53	0.087	interleukin 1 receptor, type I
Il1rap	–0.10	0.908	0.10	0.916	interleukin 1 receptor accessory protein
Il1rn	–0.31	0.783	–1.22	0.052	interleukin 1 receptor antagonist
Il6ra	–0.29	0.466	0.32	0.428	interleukin 6 receptor, alpha
Il6st	–0.18	0.645	–0.04	0.977	interleukin 6 signal transducer
Cxcl1	–0.17	0.679	–0.11	0.873	chemokine (C-X-C motif) ligand 1
Tnfrsf1a	–0.47	0.196	0.99	0.188	tumor necrosis factor receptor superfamily, member 1a
Tnfrsf1b	–0.33	0.565	0.20	0.046	tumor necrosis factor receptor superfamily, member 1b
Soleus					
Ifngr1	–0.27	0.423	–0.15	0.775	interferon gamma receptor 1
Il10rb	–0.10	0.876	0.10	0.879	interleukin 10 receptor, beta
Il6ra	–0.18	0.692	–0.12	0.845	interleukin 6 receptor, alpha
Il6st	–0.31	0.151	–0.16	0.602	interleukin 6 signal transducer
Tnfrsf1a	–0.42	0.162	0.21	0.968	tumor necrosis factor receptor superfamily, member 1a
Liver					
Ifngr1	–0.07	0.935	–0.13	0.805	interferon gamma receptor 1
Ifngr2	0.15	0.678	–0.05	0.961	interferon gamma receptor 2
Il10rb	0.00	0.999	–0.06	0.964	interleukin 10 receptor, beta
Il1b	0.07	0.985	0.07	0.986	interleukin 1 beta
Il1r1	0.31	0.799	0.79	0.243	interleukin 1 receptor, type I
Il1rap	–0.01	0.998	0.00	0.999	interleukin 1 receptor accessory protein
Il1rn	0.06	0.971	0.32	0.413	interleukin 1 receptor antagonist
Il6ra	–0.24	0.550	0.55	0.055	interleukin 6 receptor, alpha
Il6st	–0.07	0.929	0.12	0.801	interleukin 6 signal transducer
Cxcl1	0.72	0.222	1.15	0.030	chemokine (C-X-C motif) ligand 1
Tnfrsf1a	–0.42	0.162	0.21	0.968	tumor necrosis factor receptor superfamily, member 1a

^1^ C57BL/6 mice (*n* = 40) were placed on a high-fat diet (fat = 60% of total kcal) for 12 weeks and then randomly assigned to a diet supplemented with quercetin only (Q), green tea extract only (E), quercetin + green tea extract (EQ), or control (i.e., high fat diet only) for four weeks. The dosage for quercetin was 25 mg of quercetin/kg of body weight (BW) per day and green tea extract dosage was 3 mg of epigallocatechin gallate/kg BW per day. Individual genes (*n* = 27) were assessed in soleus, liver, and fat. * Significantly different than the control group (*p* < 0.05).

**Table 3:** Fold change in genes downregulated in adipose and soleus tissue compared to control by supplement groups as assessed via real-time quantitative polymerase chain reaction (qPCR) analysis.

Description	*Q*	*E*	EQ	Pathways
Adipose				
Srebf2	0.44 *	0.57	0.82	Sterol biosynthesis
Atf2	0.51 *	0.83	1.10	Transcriptional activator
Sirt1	0.40 *	0.92	0.74	Stress response
Soleus				
Srebf2	0.60	0.54 *	0.62 *	Sterol biosynthesis
Pparag	1.04	0.71	0.69 *	Fatty acid storage and Glucose metabolism
Scd1	0.44	0.97	0.40 *	Fatty Acid metabolism
Cd68	0.88	0.71	0.57 *	Promote phagocytosis and activation of macrophages
Atf2	1.23	0.62 *	0.85	Transcriptional activator

^1^ C57BL/6 mice (*n* = 40) were placed on a high-fat diet (fat = 60% of total kcal) for 12-weeks and then randomly assigned to a diet supplemented with quercetin only (Q), green tea extract only (E), quercetin + green tea extract (EQ), or control (i.e., high fat diet only) for four weeks. The dosage for quercetin was 25 mg of quercetin/kg of body weight (BW) per day and green tea extract dosage was 3 mg of epigallocatechin gallate/kg BW per day. Individual genes (*n* = 27) were assessed in soleus, liver, and fat tissue. * Significantly different than the control group (*p* < 0.05).
